# New ant records from La Réunion Island (Hymenoptera, Formicidae)

**DOI:** 10.3897/BDJ.14.e188364

**Published:** 2026-03-17

**Authors:** Dominique Carval, Floriane Jacquelin, Valérie Soti, Louis Paulin, Raphaël Parnaudeau, Brian L. Fisher, Thibault Ramage

**Affiliations:** 1 CIRAD, UPR GECO, Montpellier, France CIRAD, UPR GECO Montpellier France; 2 GECO, Univ Montpellier, CIRAD, Montpellier, France, France GECO, Univ Montpellier, CIRAD Montpellier, France France; 3 CIRAD, UPR GECO, Saint-Pierre, France CIRAD, UPR GECO Saint-Pierre France; 4 CIRAD, UPR AIDA, Montpellier, France CIRAD, UPR AIDA Montpellier France; 5 AIDA, Univ Montpellier, CIRAD, Montpellier, France AIDA, Univ Montpellier, CIRAD Montpellier France; 6 CIRAD, UPR AIDA, Saint-Pierre, France CIRAD, UPR AIDA Saint-Pierre France; 7 La Réunion, Saint-André, France La Réunion Saint-André France; 8 California Academy of Sciences, San Francisco, United States of America California Academy of Sciences San Francisco United States of America; 9 Independant entomologist, Concarneau, France Independant entomologist Concarneau France

**Keywords:** Mascarene Islands, introduced species, invasive ants, Indian Ocean islands, biodiversity inventory

## Abstract

**Background:**

La Réunion Island (Mascarene Archipelago, south-western Indian Ocean) hosts a largely introduced ant fauna, shaped by historical and ongoing human-mediated introductions. Despite previous inventories, the ant fauna of the Island remains incompletely documented and updated faunistic records are needed to refine species checklists and improve knowledge of regional biodiversity. Documenting new occurrences contributes to a better understanding of species distributions, biogeographic patterns and invasion dynamics on oceanic islands, which are particularly vulnerable to biological invasions.

**New information:**

Following recent fieldwork, we report eight new species for La Réunion Island: *Brachymymex australis* Forel, 1901; *Cardiocondyla obscurior* Wheeler, 1929; *Monomorium exiguum* Forel, 1894; *Pheidole parva* Mayr, 1865; *Solenopsis globularia* Smith, 1858; Solenopsis gr. pygmaea, Stigmatomma cf. zwaluwenburgi Williams, 1946; and *Strumigenys membranifera* Emery, 1869. All are introduced species with varying invasiveness status. This work brings the total number of ants known from La Réunion Island to 62, although the presence and identification of some species cited in literature and databases needs verification. Further collections may uncover additional introduced species in urbanised and anthropogenised habitats and native species specific to La Réunion Island or the Mascarene Islands in natural ecosystems.

## Introduction

Oceanic islands are particularly sensitive to biological invasions and their ant faunas are often dominated by introduced species. Based on the most recent published sources reporting ants from La Réunion Island, a French overseas territory in the Mascarene Archipelago (Indian Ocean Blard et al. 2003, Fisher and Bolton 2016, Mévergnies et al. 2024, AntWeb 2024, AntWiki 2024), a total of 54 ant species are currently documented from the Island. Amongst them, 41 species are introduced, whereas 11 species are native (*Camponotus hova* Forel, 1891; *Camponotus maculatus* Fabricius, 1782; *Hypoponera ludovicae* Forel, 1892; *Monomorium termitobium* Forel, 1892; *Plagiolepis madecassa* Forel, 1892; *Solenopsis mameti* Donisthorpe, 1946; *Syllophopsis cryptobia* Santschi, 1921; *Tapinoma subtile* Santschi, 1911; *Technomyrmex difficilis* Forel, 1892; *Tetramorium schaufussii* Forel, 1891; and *Tetramorium sikorae* Forel, 1892), while two species are endemic to the Mascarene Islands (*Camponotus aurosus* Roger, 1863; *Pristomyrmex browni* Wang, 2003) (Wang 2003, Bolton 2014, Janicki et al. 2016, Guénard et al. 2017). Here, we report eight ant species recorded for the first time from La Réunion Island, based on recent field surveys and verified taxonomic identifications.

## Materials and methods

### Collection sites and methods

**Figure 1.**
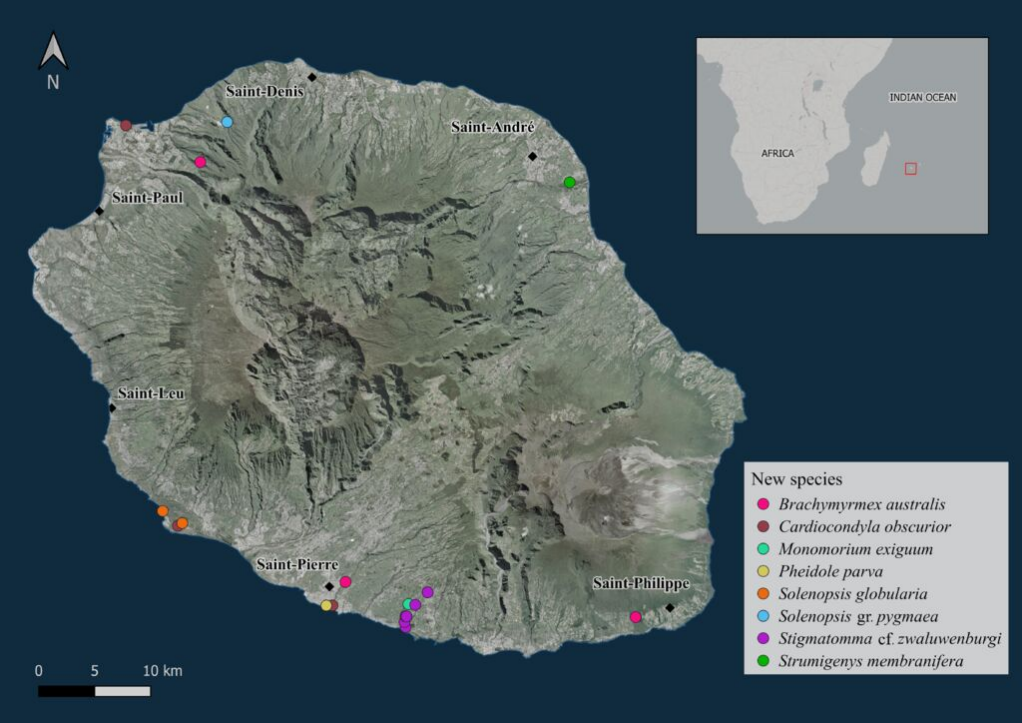
Localities of examined specimens. Base map derived from ESRI Satellite imagery (WGS84). Map produced by F. Jacquelin (CIRAD, 2024).

Sampling was performed at a variety of sites on La Réunion Island (Fig. 1). Collection methods included aspirator, forceps, interception traps, Winkler/Moczarski leaf litter sifting and beating tray. Ant samples were stored in ethanol (95%) before morphological identification.

### Morphological identification

Specimens were examined with a ZEISS Stemi 08 stereomicroscope and photographs taken with a DeltaPix Invenio EIII camera and the associated DeltaPix Insight software. Taxonomic identification was carried out first at the genus level following Fisher and Bolton (2016), then using the following keys to species: (Bolton 1987, Bolton 2000, Heterick 2006, Fischer and Fisher 2013, Pacheco and Mackay 2013, Ortiz-Sepulveda et al. 2019, Hamer et al. 2023). For *Brachymyrmex* specimens, we performed the following measurements: head width (HW), head length (HL) being the distance from the posterior margin of the frontal triangle to the vertexal margin, scape length and eye length (EL) being the maximum diameter of the compound eye and the scape length exceeding the posterior margin of the head (SLE) (Ortiz-Sepulveda et al. 2019). We then used three indices to discriminate between potential species: (i) scape index 1(SI1 = (SL/HW) × 100) (ii) scape index 2 (SI2 = (SL/HL) × 100) and (iii) scape index 3 (SI1 = EL – SLE) (Ortiz-Sepulveda et al. 2019).

### Repositories

Collections are referred to by the following acronyms:

- CAS = California Academy of Science, San Francisco, U.S.A.;
- CRP = Collection of Raphaël Parnaudeau;
- CTR = Collection of Thibault Ramage, Concarneau, France;
- CDC = Collection of Dominique Carval, Saint-Pierre, La Réunion;
- NHM = Natural History Museum of La Réunion Island;
- C3P = CIRAD Pôle de Protection des Plantes, Saint-Pierre, La Réunion.

## Taxon treatments

### Stigmatomma zwaluwenburgi

Williams, 1946

F8FF50D5-2B70-5D30-BF17-1D0FD1599608

#### Materials

- **Type status:** Other material. **Occurrence:** lifeStage: 1 gyne; occurrenceID: 5D3C472C-58DB-532D-AE22-FF2B4CB1C90E; **Location:** island: La Réunion; country: France; locality: Petite-Île; verbatimElevation: 90 m; decimalLatitude: -21.35919; decimalLongitude: 55.54150; **Identification:** identifiedBy: T. Ramage; D. Carval; **Event:** samplingProtocol: interception trap; verbatimEventDate: 22.III.2023; habitat: sugarcane field; **Record Level:** institutionCode: CAS
- **Type status:** Other material. **Occurrence:** lifeStage: 1 gyne; occurrenceID: 6E457803-4A96-5003-8C0A-14A4B98D3BA3; **Location:** island: La Réunion; country: France; locality: Petite-Île; verbatimElevation: 105 m; decimalLatitude: -21.35389; decimalLongitude: 55.74056; **Identification:** identifiedBy: D. Carval; T. Ramage; **Event:** samplingProtocol: interception trap; verbatimEventDate: 22.III.2023; habitat: sugarcane field; **Record Level:** institutionCode: CAS
- **Type status:** Other material. **Occurrence:** lifeStage: 1 gyne; occurrenceID: E4A115B6-C0A5-5FE2-977F-26C4E4CA4748; **Location:** island: La Réunion; country: France; locality: Petite-Île; verbatimElevation: 560 m; decimalLatitude: -21.35389; decimalLongitude: 55.74056; **Identification:** identifiedBy: D. Carval; T. Ramage; **Event:** samplingProtocol: interception trap; verbatimEventDate: 19.IV.2023; habitat: sugarcane field; **Record Level:** institutionCode: CAS
- **Type status:** Other material. **Occurrence:** lifeStage: 1 gyne; occurrenceID: 83B7795C-8243-5C12-A94E-EFA81B7D162A; **Location:** island: La Réunion; country: France; locality: Petite-Île; verbatimElevation: 198 m; decimalLatitude: -21.35331; decimalLongitude: 55.54297; **Identification:** identifiedBy: D. Carval; T. Ramage; **Event:** samplingProtocol: interception trap; verbatimEventDate: 04.I.2023; habitat: sugarcane field; **Record Level:** institutionCode: C3P; collectionCode: MGAU05446_010
- **Type status:** Other material. **Occurrence:** lifeStage: 1 gyne; occurrenceID: 2AF3E782-38BA-559B-B408-1CBB82E8D184; **Location:** island: La Réunion; country: France; locality: Petite-Île; verbatimElevation: 200 m; decimalLatitude: -21.35322; decimalLongitude: 55.54219; **Identification:** identifiedBy: D. Carval; T. Ramage; **Event:** samplingProtocol: interception trap; verbatimEventDate: 06.XII.2023; habitat: sugarcane field; **Record Level:** institutionCode: NHM
- **Type status:** Other material. **Occurrence:** lifeStage: 1 gyne; occurrenceID: D4272B29-E7FE-5CB7-AAE1-DEFFC30D593F; **Location:** island: La Réunion; country: France; locality: Petite-Île; verbatimElevation: 198 m; decimalLatitude: -21.35331; decimalLongitude: 55.54297; **Identification:** identifiedBy: D. Carval; T. Ramage; **Event:** samplingProtocol: interception trap; verbatimEventDate: 03.I.2024; habitat: sugarcane field; **Record Level:** institutionCode: CAS
- **Type status:** Other material. **Occurrence:** lifeStage: 1 gyne; occurrenceID: E65F5646-C6EB-5D9E-AAE8-CF9EA0078ECE; **Location:** island: La Réunion; country: France; locality: Petite-Île; verbatimElevation: 378 m; decimalLatitude: -21.34378; decimalLongitude: 55.54950; **Identification:** identifiedBy: D. Carval; T. Ramage; **Event:** samplingProtocol: interception trap; verbatimEventDate: 28.II.2024; habitat: sugarcane field; **Record Level:** institutionCode: CAS

#### Distribution

**Figure 2.**
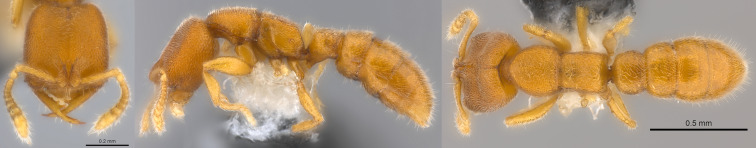
Full-face, lateral and dorsal view of a *Stigmatomma zwaluwenburgi* worker (photographer: Eli M. Sarnat, CASENT0187702 https://www.antweb.org/).

To our knowledge, the specimens from La Réunion are the first gynes collected for this species from the Malagasy Region. As the gyne of *S. zwaluwenburgi* is not known, we cannot be certain the gynes collected in Réunion Island belong to this species, but they match well with workers of *S. zwaluwenburgi* (Fig. 2). This species has been reported from very few places: Hawaii (Williams 1946), Christmas Island (Framenau and Thomas 2008), Fiji (Sarnat 2009) and Hong Kong (Hamer et al. 2023). The native range of this species remains a conundrum, but an east Asian origin seems likely (Ward and Fisher 2016, Hamer et al. 2023).

#### Ecology

The species has been very rarely collected and its ecology is little known. As for our specimens, the type material of *S. zwaluwenburgi* was collected within a sugar cane field (Williams 1946). The specimens from Fiji were collected in the litter of a disturbed forest (AntWeb 2024) and Hamer et al. (2023) collected specimens from Signal Hill Gardens, an urbanised area of Hong Kong. This cryptobiotic ant is a soil-dwelling species that tolerates anthropogenised habitats.

### Brachymyrmex australis

Forel, 1909

8E1CCCC5-8E08-57D2-BF59-4711DA8106D7

#### Materials

- **Type status:** Other material. **Occurrence:** lifeStage: 4 workers; occurrenceID: CDA9844B-B8A4-5177-894F-9B8D03E3FC6C; **Location:** island: La Réunion; country: France; municipality: Saint-Pierre; locality: Bassin Plat; verbatimElevation: 152 m; decimalLatitude: -21.32356; decimalLongitude: 55.49042; **Identification:** identifiedBy: D. Carval; T. Ramage; **Event:** samplingProtocol: aspirator; verbatimEventDate: 27.VII.2020; habitat: living tree trunk; **Record Level:** institutionCode: CDC
- **Type status:** Other material. **Occurrence:** lifeStage: 13 workers 6 males; occurrenceID: 0B17DC5F-6F69-5CE0-B538-C224E1A24FCF; **Location:** island: La Réunion; country: France; municipality: Saint-Philippe; locality: Mare Longue forest; verbatimElevation: 233 m; decimalLatitude: -21.35389; decimalLongitude: 55.74056; **Identification:** identifiedBy: D. Carval; T. Ramage; **Event:** samplingProtocol: aspirator; verbatimEventDate: 25.IV.2023; habitat: rotten wood trunk; **Record Level:** institutionCode: CDC
- **Type status:** Other material. **Occurrence:** lifeStage: 7 workers; occurrenceID: F325AA5D-69FF-5A65-9A83-44D0B85F6E7C; **Location:** island: La Réunion; country: France; municipality: La Possession; locality: Crête solitude; verbatimElevation: 670 m; decimalLatitude: -21.96178; decimalLongitude: 55.36500; **Identification:** identifiedBy: D. Carval; T. Ramage; **Event:** samplingProtocol: beating tray; verbatimEventDate: 28.III.2024; habitat: shrubs; **Record Level:** institutionCode: CDC

#### Diagnosis

**Figure 3.**
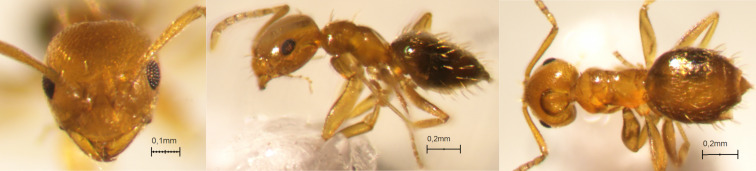
Full-face, lateral and dorsal view of a *Brachymyrmex australis* worker (photographer: D. Carval).

According to Ortiz-Sepulveda et al. (2019), *B. australis* is very similar to *B. aphidicola* Forel, 1909, but can be distinguished by scape indices 1, 2 and 3 (hereafter SI-I3). However, of our 13 specimens from the three locations, the SI1 identified none as *B. australis*, three as *B. aphidicola* and led 10 times to an uncertain classification (Table 1). Similarly, SI2 identified six specimens as *B. australis*, none as *B. aphidicola* and led seven times to an uncertain classification (Table 1). By contrast, SI3 identified all the specimens as *B. australis*. On La Réunion Island, this ant can be confused with *Plagiolepis alluaudi* Emery, 1894 from which it can be distinguished by the number of antennal segments (nine for *B. aphidicola*, 11 for *P. alluaudi*). *Brachymyrmex australis* can be distinguished from *Brachymyrmex cordemoyi* Forel, 1895 by its colouration and worker size polymorphism. *Brachymyrmex australis* typically exhibits a uniformly light orange colouration (Fig. 3), whereas *B. cordemoyi* is generally dark brown, although lighter individuals may occur. In addition, workers of *B. australis* are monomorphic in size, while those of *B. cordemoyi* show marked size polymorphism.

#### Distribution

This species is native to South America and Central America and has been reported as introduced in Mauritius (Ortiz-Sepulveda et al. 2019, Rosas-Mejía et al. 2021).

#### Ecology

This species has been found under stones and in rotten wood and epiphytes (Ortiz-Sepulveda et al. 2019). *B. australis* nests underground, in organic litter (Wild 2007) or in rotting tree trunks

### Pheidole parva

Mayr, 1865

920A7FF5-F591-50CD-B5F7-F65FA322125E

#### Materials

- **Type status:** Other material. **Occurrence:** individualCount: 10 workers; occurrenceID: 4ABBD950-E963-59C3-8D54-B3AC14F968DB; **Location:** island: La Réunion; country: France; municipality: Saint-Pierre; locality: Front de mer; verbatimElevation: 6 m; decimalLatitude: -21.34389; decimalLongitude: 55.47389; **Identification:** identifiedBy: D. Carval; **Event:** samplingProtocol: pincers; verbatimEventDate: 03.I.2024; habitat: beach front; **Record Level:** collectionCode: CDC
- **Type status:** Other material. **Occurrence:** individualCount: 3 minor workers, 1 major worker; occurrenceID: 58F1D2AB-A268-59E6-A570-BAC64EA6DDCC; **Location:** island: La Réunion; country: France; municipality: Saint-Pierre; locality: Front de mer; verbatimElevation: 6 m; decimalLatitude: -21.34389; decimalLongitude: 55.47389; **Identification:** identifiedBy: D. Carval; **Event:** samplingProtocol: pincers; verbatimEventDate: 03.I.2024; habitat: beach front; **Record Level:** collectionCode: CDC

#### Diagnosis

**Figure 4.**
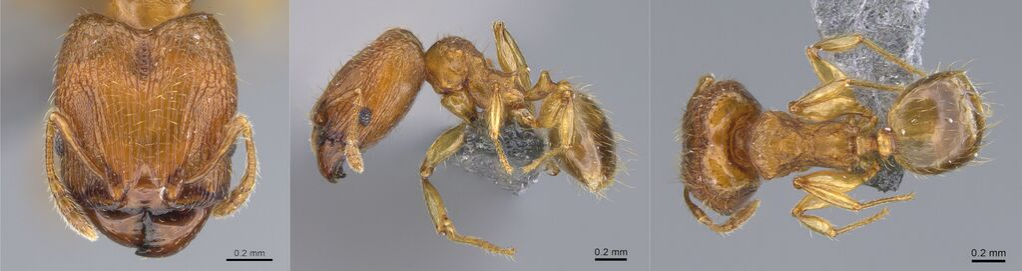
Full-face, lateral and dorsal view of a *Pheidole parva* major worker (photographer: April Nobile, CASENT0055997 from  https://www.antweb.org/).

**Figure 5.**
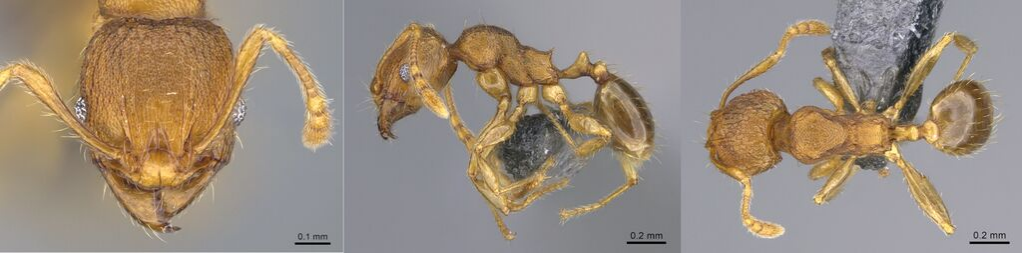
Full-face, lateral and dorsal view of a *Pheidole parva* minor worker (photographer: April Nobile, CASENT0055998 from  https://www.antweb.org/).

*Pheidole parva* can be distinguished from the other *Pheidole* species present in La Réunion Island (*Pheidole megacephala* (Fabricius, 1793) by its smaller size, the fact that both minors and majors are prominently sculptured, head of majors more elongate and with a broadly V-shaped posterior emargination and postpetiole without a large convex ventral process (Fischer and Fisher 2013) (Figs 4, 5). In Mauritius, two other introduced *Pheidole* species are reported (*Pheidole fervens* Smith, 1858 and *Pheidole indica* Mayr, 1879) and it would not be surprising if they are found on La Réunion Island as well (Fischer and Fisher 2013).

#### Distribution

This species is native to the Indomalaya Region and has been reported as introduced in Japan, Mauritius, Rodrigues, Seychelles, Oman and Saudi Arabia. It has also been reported from indoor locations in Germany and Austria.

#### Ecology

This invasive species is well adapted to human environments, such as ports and urban beachfronts. This ant forages or nests in various habitats such as soil, litter, under stones, in root mats, rotten logs or the bark of live trees. This species likely invades new areas through human commerce, especially maritime commerce (Fischer and Fisher 2013).

### Strumigenys membranifera

Emery, 1869

87211A88-FD99-5F42-B690-1722DB0CD5B5

#### Materials

- **Type status:** Other material. **Occurrence:** individualCount: 3 workers; occurrenceID: 5C397122-D41A-59C2-9B64-B12DBAB58E7B; **Taxon:** genus: Strumigenys; specificEpithet: *membranifera*; scientificNameAuthorship: Emery, 1869; **Location:** island: La Réunion; country: France; municipality: Saint-André; locality: Front de mer; verbatimElevation: 21 m; decimalLatitude: -20.97917; decimalLongitude: 55.68361; **Identification:** identifiedBy: T. Ramage; **Event:** verbatimEventDate: 10.XII.2023; habitat: beach front; **Record Level:** collectionID: CRP

#### Diagnosis

**Figure 6.**
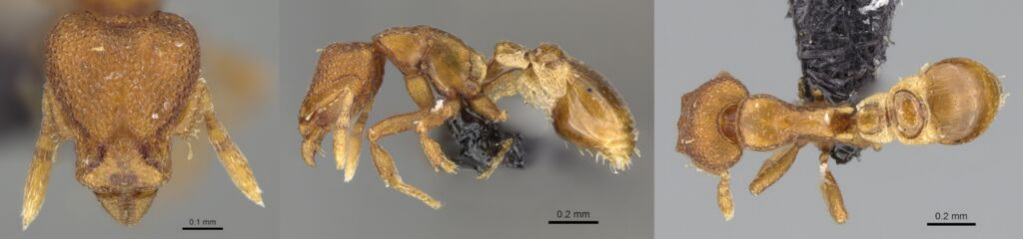
Full-face, lateral and dorsal view of a *Strumigenys membranifera* worker (photographer: Michele Esposito, CASENT0023769 from  https://www.antweb.org/).

This species can be confused with the four other *Strumigenys* species present in La Réunion Island (*Strumigenys emmae* (Emery, 1890), *Strumigenys ludovici* (Forel, 1904), *Strumigenys nepalensis* (De Andrade, 1994), *Strumigenys rogeri* Emery, 1890). *S. membranifera* can be differentiated from *S. emmae* and *S. rogeri* by its distinctive mandibular shape. In *S. membranifera*, the mandibles are triangular and short, featuring numerous teeth along the inner margin (Fig. 6). In contrast, *S. rogeri* displays elongated and slender mandibles, while *S. emmae* exhibits sickle-shaped mandibles, both of which possess apical and pre-apical teeth. It differs from *S. nepalensis* by the number of antennal segments (six for *S. membranifera*, four for *S. nepalensis*). Finally, *S. membranifera* has shorter mandibles with fewer teeth (< 20) and a more rounded head, while *S. ludovici* has longer mandibles with more than 25 teeth and a more elongate head than *S. membranifera*.

#### Distribution

Besides *S. emmae* and *S. rogeri*, this species is one of the most widespread dacetine ants in the world (Wetterer 2011). This highly invasive species is considered native to several countries of Africa, although phylogenetic analyses place the origin of *S. membranifera* in the Indomalay Region (Booher et al. 2021). This species is widespread worldwide and reported as introduced in South America, United States of America, Europe, Asia, Oceania and the Malagasy Region.

#### Ecology

As with other dacetines, *S. membranifera* is a predatory species that primarily feeds on springtails (Collembola) and other minuscule soil arthropods (Wilson 1953). Typically dwelling within soil and leaf litter, *S. membranifera* seldom ventures openly above ground for foraging purposes (Wetterer 2011). Thanks to minuscule teeth on pliers-like mandibles, *S. membranifera* can clamp down on and securely hold prey (Wetterer 2011).

### Cardiocondyla obscurior

Wheeler, 1929

52D5F8EE-EAA6-5F3F-82CE-7EFFB67D2F42

#### Materials

- **Type status:** Other material. **Occurrence:** individualCount: 6 workers; occurrenceID: A023853A-0DCE-51C1-8985-EA38D88E9595; **Taxon:** genus: Cardiocondyla; specificEpithet: *obscurior*; scientificNameAuthorship: Wheeler, 1929; **Location:** island: La Réunion; country: France; municipality: Saint-Pierre; locality: Front de mer; verbatimElevation: 8 m; decimalLatitude: -21.34389; decimalLongitude: 55.47917; **Identification:** identifiedBy: D. Carval; **Event:** samplingProtocol: pincers; verbatimEventDate: 06.VI.2024; habitat: beach front; **Record Level:** collectionID: CDC

#### Diagnosis

**Figure 7.**
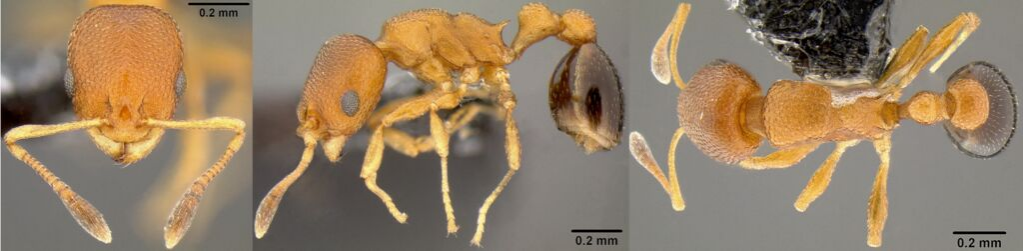
Full-face, lateral and dorsal view of a *Cardiocondyla obscurior* worker (photographer: Eli M. Sarnat, CASENT0171038 from  https://www.antweb.org/ ) .

This species closely resembles *Cardiocondyla wroughtonii* (Forel, 1890), which has been reported from La Réunion Island. Workers of *C. obscurior* (Fig. 7) differ from *C. wroughtonii* in having a darker gaster, a shorter head, a reduced postocular distance, a narrower frons, broader and higher waist segments, a greater distance between the bases of the propodeal spines and shorter propodeal spines (Seifert 2002).

#### Distribution

This species is native to the Indomalaya Region and has been reported as introduced in many locations, such as Brazil (Meurer et al. 2015), Venezuela (Guénard et al. 2017, Janicki et al. 2016), France and Belgium (Blatrix et al. 2018), Spain (Sánchez-García and Espadaler 2015) and Kenya (Garcia et al. 2013).

#### Ecology

*Cardiocondyla obscurior* has been reported from a variety of disturbed and undisturbed habitats, such as urban areas, grasslands, agricultural areas and forest margins (Seifert 2002). Nests are typically located in cavities of bushes and trees, but the species has also been found in plant galls and dead twigs.

### Monomorium exiguum

Forel, 1894

97E72725-28AC-537E-A01A-3FDEC062BE1A

#### Materials

- **Type status:** Other material. **Occurrence:** individualCount: 1 worker; occurrenceID: 536F5F1A-9029-58A4-A5F4-FF17AF2C0BE5; **Taxon:** genus: Monomorium; specificEpithet: *exiguum*; scientificNameAuthorship: Forel 1894; **Location:** island: La Réunion; country: France; municipality: Petite Île; verbatimElevation: 378 m; decimalLatitude: -21.34378; decimalLongitude: 55.54950; **Identification:** identifiedBy: D. Carval; **Event:** samplingProtocol: interception trap; verbatimEventDate: 19.XII.2023; habitat: sugarcane field; **Record Level:** collectionID: CDC

#### Diagnosis

**Figure 8.**
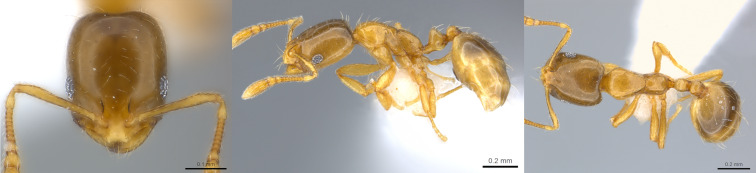
Full-face, lateral and dorsal view of a *Monomorium exiguum* worker (photographer: Erin Prado, CASENT0217367 from https://www.antweb.org/).

Amongst the 20 *Monomorium* species reported in the Malagasy Region (Fisher and Bolton 2016), *M. exiguum* is easily distinguished by its 11-segmented antennae (*M. monomorium* species group), a metanotal groove weakly impressed, an elongated propodeum and a petiolar node that is conical and dorsally tapered (Heterick 2006) (Fig. 8). All other *Monomorium* species reported from La Réunion Island have 12-segmented antennae (*Monomorium floricola* (Jerdon, 1851), *Monomorium pharaonis* (Linnaeus, 1758), *Monomorium subopacum* (Smith, 1858) and *Monomorium termitobium* (Forel, 1892)). This species displays great variability in colour, from yellow pale to brown (Heterick 2006), but our specimen was a pale orange similar to that found in *M. exiguum flavescens* (Forel, 1916), now considered as a junior synonym of *M. exiguum* (Bolton 2014).

#### Distribution

This species is native to the Afrotropical Region, where it is widely distributed in sub-Saharan Africa, including Madagascar (AntWeb 2024) and the Arabian Peninsula (Aldawood and Sharaf 2009). It has been reported as introduced on the Socotra Archipelago (Sharaf et al. 2018), in the Balearic Islands (Gómez and Espadaler 2006) and in Crete (Borowiec et al. 2023). This is the first report of this species in the Mascarene Islands.

#### Ecology

*Monomorium exiguum* appears adapted to numerous types of habitats. In the Arabian Peninsula, it has been found in humid soil, in leaf litter, under rocks and under the bark of trees (Sharaf et al. 2018). Interestingly, it has been found inside the galleries of a *Camponotus* species (Sharaf et al. 2018) and inside the galleries of a termite nest (Sharaf et al. 2017). In the Province of Tolaria in Madagascar, *M. exiguum* has been found to be the most abundant *Monomorium* species in leaf litter samples (Heterick 2006). In Benin, one specimen was found in a mango orchard with no specification of the microhabitat (Taylor et al. 2018). In La Réunion Island, the specimen was found in a sugarcane field.

### Solenopsis globularia

Smith, 185

AF4641EB-FE59-5018-836B-5DC883E17CEA

#### Materials

- **Type status:** Other material. **Occurrence:** individualCount: 2 workers; occurrenceID: 1A1DDE49-A486-54B8-9D37-2FF4A2287845; **Taxon:** genus: Solenopsis; specificEpithet: *globularia*; scientificNameAuthorship: Smith, 1858; **Location:** island: La Réunion; country: France; municipality: Etang-Salé; locality: Piton Reinette; verbatimElevation: 53 m; decimalLatitude: -21.27444; decimalLongitude: 55.34750; **Identification:** identifiedBy: D. Carval, T. Ramage; **Event:** samplingProtocol: aspirator; verbatimEventDate: 25.IV.2023; habitat: litter; **Record Level:** collectionID: CDC
- **Type status:** Other material. **Occurrence:** individualCount: 2 workers; occurrenceID: 3BF9971A-F882-5C5A-A268-CB6838C68BEB; **Taxon:** genus: Solenopsis; specificEpithet: *globularia*; scientificNameAuthorship: Smith, 1858; **Location:** island: La Réunion; country: France; municipality: Etang-Salé; locality: Beach front; verbatimElevation: 11 m; decimalLatitude: -21.26250; decimalLongitude: 55.33278; **Identification:** identifiedBy: D. Carval, T. Ramage; **Event:** samplingProtocol: aspirator; verbatimEventDate: 25.IV.2023; habitat: beach front; **Record Level:** collectionID: CDC

#### Diagnosis

**Figure 9.**
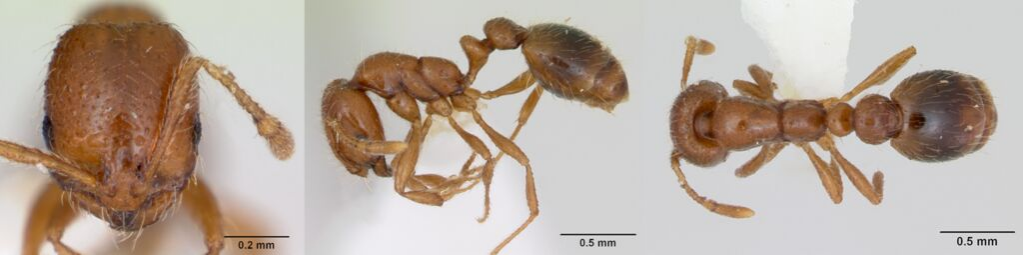
Full-face, lateral and dorsal view of a *Solenopsis globularia* worker (photographer: April Nobile, CASENT0173279 from https://www.antweb.org/).

This species can be confused with the two other *Solenopsis* species (*Solenopsis geminata* (Fabricius, 1804) and *Solenopsis mameti* Donisthorpe, 1946) as well as with the three *Cardiocondyla* species reported from La Réunion Island: *Cardiocondyla emeryi* Forel, 1881, *Cardiocondyla itsukii* and *Cardiocondyla wroughtonii* (Forel, 1890). It differs from other *Solenopsis* by its enlarged postpetiole and from *Cardiocondyla* species by its 2-segmented antennal club and the absence of propodeal spines (Fig. 9).

#### Distribution

This introduced species is native to south-eastern regions of the United States, Caribbean, Central America and South America. It has been introduced to several African countries and islands, as well as the Philippines and Oceania (French Polynesia, Galápagos, Hawaii) (Pacheco and Mackay 2013).

#### Ecology

According to Wetterer (2019), this intermediate-sized invasive species behaves more like a small fire ant rather than a thief ant. This species forages on the ground and may be collected at bait traps and nests under rocks and logs (Pacheco and Mackay 2013).

### Solenopsis pygmaea

Forel, 1901

450672E2-7C36-5B50-9D06-049175C3DBAA

#### Materials

- **Type status:** Other material. **Occurrence:** individualCount: 2 workers; occurrenceID: 6E4F2A93-F0F2-57C7-9F2C-B45D4DE20128; **Location:** island: La Réunion; country: France; municipality: La Possession; locality: Crête Ti Bon Dieu; verbatimElevation: 534 m; decimalLatitude: -20.92444; decimalLongitude: 55.38833; **Identification:** identifiedBy: D. Carval, T. Ramage; **Event:** samplingProtocol: pincers; verbatimEventDate: 29.III.2024; habitat: rotten wood trunk; **Record Level:** collectionID: CDC
- **Type status:** Other material. **Occurrence:** individualCount: 3 workers; occurrenceID: BA968C8F-B8D4-5609-B8FB-A44F1F6CCEE1; **Location:** island: La Réunion; country: France; municipality: La Possession; locality: Crête Ti Bon Dieu; verbatimElevation: 534 m; decimalLatitude: -20.92444; decimalLongitude: 55.38833; **Identification:** identifiedBy: D. Carval, T. Ramage; **Event:** samplingProtocol: Winkler-Moczarski; verbatimEventDate: 29.III.2024; habitat: rotten wood trunk; **Record Level:** collectionID: CDC

#### Diagnosis

**Figure 10.**
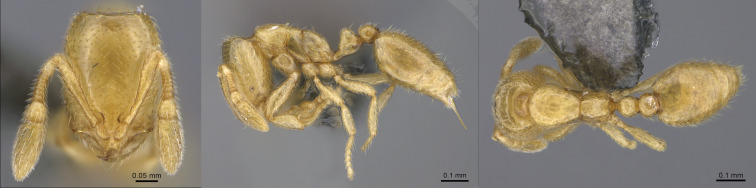
Full-face, lateral and dorsal view of a Solenopsis gr. pygmaea worker (photographer: Michele Esposito, CASENT0923738 from  https://www.antweb.org/).

This species is easily distinguishable from the three other *Solenopsis* species reported from La Réunion Island (Fig. 10). Firstly, this thief ant species is significantly smaller than *S. geminata* (Fabricius, 1804) and *S. globularia* with a total length < 1.2 mm. The second thief ant species present on the Island, *S mameti*, exhibits uniformly dark body colouration, while Solenopsis gr pygmaea is concolorous pale yellow.

#### Distribution

To date, the identification to species has not been confirmed. This species has been compared to Afrotropical and Australasian *Solenopsis*, but does not match any species. This *Solenopsis* seems to have an affinity with the neotropical *Solenopsis*, confirmed by the barcoding of the Mauritius specimens that share a BIN (BOLD:AAC3883) with specimens from Bahamas, Costa Rica, Puerto Rico and Suriname. This *Solenopsis* was likely introduced to the Mascarene Islands, despite the fact that it was found in a natural habitat on La Réunion Island.

#### Ecology

This species has been found nesting in rotten wood and litter. As with other thief ants, this species probably has lestobiotic and cryptic habits (Pacheco and Mackay 2013).

## Discussion

With the discovery of these eight new reported species, the total number of ant species documented on La Réunion Island now stands at 62 (AntWeb 2024, Mévergnies et al. 2024). These surveys suggest that other introduced species may remain undiscovered on the Island. Similar to past invasions, we can theorise that introductions are associated with maritime or air cargo. These introductions might also arise from the movement of plant materials or soil substrates by travellers. Meanwhile, the total number of species recorded on Mauritius Island is 78, including 11 species endemic to the Island or the Mascarene Islands (AntWeb 2024). Only two endemic species of the Mascarene Islands (*Camponotus aurosus* and *Pristomyrmex browni*) are reported from La Réunion Island, despite its significantly larger area of preserved primary forests compared to Mauritius. Therefore, further studies are needed in both urbanised and human-impacted environments to identify more introduced species, as well as in natural habitats to discover species endemic to La Réunion Island or the Mascarene Islands. Finally, the origin of the new reported species should be investigated using molecular techniques, such as COI barcoding.

## Supplementary Material

XML Treatment for Stigmatomma zwaluwenburgi

XML Treatment for Brachymyrmex australis

XML Treatment for Pheidole parva

XML Treatment for Strumigenys membranifera

XML Treatment for Cardiocondyla obscurior

XML Treatment for Monomorium exiguum

XML Treatment for Solenopsis globularia

XML Treatment for Solenopsis pygmaea

## Figures and Tables

**Table 1.** Morphological measurements (mm) on 14 *Brachymyrmex* specimens. According to Ortiz-Sepulveda et al. (2019): if SI1 < 94 or SI1 > 104, the *Brachymyrmex* specimen is classified as *B. australis* or *B. aphidicola*, respectively; if 94 ≤ SI1 ≤ 104, the classification of the specimen is considered uncertain. Similarly, if SI2 < 117 or SI2 > 135, the *Brachymyrmex* specimen is classified as *B. australis* or *B. aphidicola*, respectively; f 117 ≤ SI2 ≤ 135, the classification of the specimen is considered uncertain. Finally, if SI3 ≥ 0, the *Brachymyrmex* specimen is classified as *B. australis*; otherwise, it is classified as *B. aphidicola*. Specimen 1 was sampled at 21°19'24.8"S, 55°29'25.5"E, specimens 2–7 were sampled at 21°21'14"S, 55°44'26"E and specimens 8–13 were sampled at 20°57'42.4"S 55°21'54.6"E.

**Specimen**	**HL**	**HW**	**EL**	**SLE**	**SI1**	**SI2**	**SI3**	**Classification (SI1)**	**Classification (SI2)**	**Classification (SI3)**
1	0.32	0.38	0.09	0.08	110.5	131.3	0.01	*B. aphidicola*	*uncertain*	*B. australis*
2	0.34	0.38	0.10	0.09	105.3	117.6	0.01	*B. aphidicola*	*uncertain*	*B. australis*
3	0.35	0.40	0.10	0.06	102.5	117.1	0.04	*uncertain*	*uncertain*	*B. australis*
4	0.35	0.39	0.10	0.06	102.6	114.3	0.04	*uncertain*	*B. australis*	*B. australis*
5	0.34	0.39	0.09	0.05	100.0	114.7	0.04	*uncertain*	*B. australis*	*B. australis*
6	0.34	0.40	0.10	0.05	95.0	111.8	0.05	*uncertain*	*B. australis*	*B. australis*
7	0.32	0.38	0.10	0.04	97.4	115.6	0.06	*uncertain*	*B. australis*	*B. australis*
8	0.32	0.41	0.11	0.08	100.0	128.1	0.03	*uncertain*	*uncertain*	*B. australis*
9	0.34	0.41	0.10	0.05	95.1	114.7	0.05	*uncertain*	*B. australis*	*B. australis*
10	0.32	0.40	0.10	0.07	102.5	128.1	0.03	*uncertain*	*uncertain*	*B. australis*
11	0.33	0.39	0.09	0.06	100.0	118.2	0.03	*uncertain*	*uncertain*	*B. australis*
12	0.33	0.37	0.10	0.07	105.4	118.2	0.03	*B. aphidicola*	*uncertain*	*B. australis*
13	0.33	0.37	0.08	0.04	102.7	115.2	0.04	*uncertain*	*B. australis*	*B. australis*
